# Erythrocyte concentrations of chromium, copper, manganese, molybdenum, selenium and zinc in subjects with different physical training levels

**DOI:** 10.1186/s12970-020-00367-4

**Published:** 2020-07-09

**Authors:** M. Maynar, F. J. Grijota, J. Siquier-Coll, I. Bartolome, M. C. Robles, D. Muñoz

**Affiliations:** 1 School of Sport Sciences, University of Extremadura, University Avenue, Avenida de la Universidad s/n, 10003 Cáceres, Spain; 2 School of Education, University of Extremadura, University Avenue, s/n, 10003 Cáceres, Spain

**Keywords:** Essential trace elements, Red blood cells, Exercise, Adaptations

## Abstract

**Background:**

The aim of the present study was to determine changes occurring in the erythrocyte concentrations of chromium (Cr), copper (Cu), manganese (Mn), molybdenum (Mo), selenium (Se) and zinc (Zn) in male subjects with different training levels living in the same region (Spain).

**Methods:**

Thirty sedentary subjects (24.34 ± 3.02 years) formed the control group (CG); 24 moderately trained (4–7 h/week) subjects (23.53 ± 1.85 years) formed the group with a moderate degree of training (MTG) and 22 professional cyclists (23.29 ± 2.73 years), who performed more than 20 h/week of training, formed the high-level training group (HTG). Erythrocyte samples were collected from all subjects in fasting conditions, washed and frozen at − 80 °C until analysis. Erythrocyte analysis of trace elements was performed by inductively coupled plasma mass spectrometry (ICP-MS).

**Results:**

The results showed that there was a statistically significant lower erythrocyte concentration of Cu, Mn, Mo and Zn in the MTG and HTG than CG. Se was only significantly lower in HTG than CG. The correlation analysis indicates that this change was correlated with training in the case of Cu, Mn, Se and Zn. All results are expressed in μg/g Hb.

**Conclusions:**

We can conclude that physical training produces a decrease in erythrocyte concentrations of Cu, Mn, Se and Zn, which can cause a decrement in athletes’ performance given the importance of these elements. For this reason, erythrocyte monitoring during the season would seem to be advisable to avoid negative effects on performance.

## Introduction

Micronutrients, trace minerals and vitamins are integral components of many metabolic processes and are required in minimal amounts in the daily diet to maintain health. Deficiencies in micronutrients (especially in phosphorus and magnesium) are prevalent in developing countries (first-world adult populations) and can constitute a significant health problem, leading, for example, to diabetes, cardiovascular and kidney disease, aging and fracture risk [1]. Population data suggest that current recommended dietary allowance are not being achieved, with selenium, magnesium, calcium, iron and zinc of particular concern [2].

The body concentration of micronutrients is usually determined by measuring the trace elements in plasma; however, the plasma concentration of these elements may fluctuate regardless of nutritional status [3]. Previous studies have observed a decrease in erythrocyte concentrations of other macro minerals, such as magnesium, phosphorus and iron in athletes, compared with a control group [4]. However, very few studies have determined the erythrocyte concentrations of trace elements, and the influence of physical training.

Cr is related to numerous enzymatic processes. It can affect muscle activity and lipid profile positively, increasing the number of receptors for insulin [5, 6]. A recent study observed increases in serum concentrations of Cr during exercise. This would increase glucose and insulin activity and therefore glucose use, and could contribute positively to the body fat profile by decreasing cholesterol and triglyceride levels [7].

Cu is also a crucial micronutrient involved in energy metabolism and the antioxidant system [8], which prevents or reduces the effects of an excessive production of reactive oxygen species. In addition, in humans, it is needed for proper organ function and metabolic processes such as the formation of hemoglobin [9]. Zinc (Zn) is an essential nutrient for living organisms and their biological processes [10]. The body cannot accumulate zinc and it is, therefore, essential to take this element consistently in the diet [11]. During the inflammation occurring in response to oxidative damage, the plasma concentrations of Zn and Se fall while those of Cu rise; plasma Se and Zn are partially bound to albumin and can be influenced by its plasma concentrations. The concentration in plasma, therefore, can be affected by other factors that make its interpretation difficult [3]. The cytokines produced during the inflammatory response increase the permeability of capillaries, to redistribute in the interstitium, to albumin and other proteins [12]. Other decreases in the zinc concentration during an acute inflammatory response arise from the sequestration of zinc in the liver and other tissues [13]. The level of Cu rises since ceruloplasmin, the protein that contains the most Cu, increases in the acute phase of inflammation [14].

Few studies have been found about the effects of exercise on Mn concentrations. However, it is a crucial trace element of an antioxidant enzyme, superoxide dismutase (SOD), which converts the superoxide radical to hydrogen peroxide (H_2_O_2_). A recent study observed higher values of Mn-SOD in exercisers than sedentary people, that was correlated with vigorous exercise [15]. Another study that investigated the time course of exercise induction of SOD in human skeletal muscle found elevated levels of SOD after exercise [16].

Finally, Mo is a trace element involved in several redox processes, being part of enzymatic systems. Contradictory results have been observed previously in relation to the effect of physical activity. No changes have been found in plasma and urine concentrations of this mineral after a marathon or an incremental test until exhaustion [17, 18].

For all the above, the intracellular determination of these elements has been proposed, more specifically in erythrocytes [19], as the erythrocyte concentration of trace elements is not affected by the acute inflammatory response [19] or by the short-term diet. Therefore, the aim of the study was to ascertain the intraerythrocytic concentrations of Cr, Cu, Mn, Mo, Se and Zn in sedentary men, and others with a moderate and high degree of physical training given the importance that possible deficits can have for their sports performance, and if such concentrations can depend on the degree of training.

## Materials and methods

The materials and methods are the same as described previously in Maynar-Mariño et al., (2020) [4]. However, they are explained again below.

### Participants

Thirty sedentary subjects aged 24.34 ± 3.02 years without sport practice and a less active lifestyle formed the control group (CG). Twenty-four non-professional subjects aged 23.53 ± 1.85 years, who perform between 4 and 7 h/week of moderate sport practice without any performance objective, and without following any systematic training, formed the group with a moderate degree of training (MTG). Twenty-two high-level professional cyclists aged 23.29 ± 2.73, at the beginning of their sport season, who performed more than 20 h/week of training, formed the high-level training group (HTG). On the basis of the total hours/week of physical activity, the subjects were classified into three categories: low (CG), moderate (MTG) and high (HTG).

Each participant had to satisfy the following criteria in order to be included in the study: to be a man, a non-smoker and not to have any health problems. The participants could not take any vitamins, minerals or other supplements during the study.

Subjects were informed about the aim and procedures of the study, gave their informed consent and participated voluntarily. The University of Extremadura Ethics Committee approved the investigation according to the latest version of the Helsinki declaration for human research.

### Anthropometric measurements

The morphological characteristics of the participants were measured in the morning and always at the same time and under identical conditions. Body height was measured to the nearest 0.1 cm using a wall-mounted stadiometer (Seca 220. Hamburg. Germany). Body weight was measured to the nearest 0.01 kg using calibrated electronic digital scales (Seca 769. Hamburg. Germany) in nude, barefoot conditions. Body fat content was estimated from the sum of 6 skinfolds (∑6) (abdominal, suprailiac, tricipital and subscapularis, thigh and calf skinfolds). The skinfold thicknesses were measured with a Harpenden caliper (Holtain Skinfold Caliper. Crosswell, UK). All measurements were made by the same operator, skilled in kinanthropometry techniques, following the International Society for the Advancement of Kinanthropometry recommendations. Heart rate and blood pressure were determined using an automatic sphygmomanometer (Omron HEM-780. Osaka. Japan) by a skilled technician, always after a five-minute rest period in the supine position.

### Nutritional evaluation

To guarantee they were following a similar diet, all participants completed a dietary questionnaire which consisted of a 3-day daily nutritional record, on two pre-assigned weekdays and one weekend day. On each day, participants individually indicated the type, frequency and quantity (in grams) of every food consumed, and then the nutritional composition of their diets was evaluated using different food composition tables [20–22].

### Incremental test until exhaustion

An exercise test was used to evaluate the performance variables. The test consisted of a progressive load until exhaustion, on a cycle ergometer (Ergoline 900; Bitz, Germany) equipped with a gas analyzer (Metamax. Cortex Biophysik. Gmbh. Germany) and a Polar pulsometer (Polar. Norway).

Depending on the degree of training, two different protocols were used. The exercise protocol used for the HTG consisted of 1 min entirely at rest (to measure HR at rest), 15 min of warm-up, ending with 5 min at 100 watts; then starting at 150 watts and increasing the intensity by 25 watts every 3 min until reaching the maximum power they could maintain. In the case of MTG and CG, it consisted of 1 min entirely at rest, 15 min of warm-up ending with 5 min at 40 watts. Then, starting at 50 watts and increasing the intensity by 25 watts every 3 min until voluntary exhaustion (VO_2_ max), reaching the maximum power they could produce (the last number of watts attained was considered the maximum power). All tests were carried out under similar atmospheric conditions (21–24 °C and 45–55% relative humidity and atmospheric pressure between 700 and 715 mmHg).

The choice of these protocols was based on previous studies in which a slight increase in intensity was recommended in each step [23] and an adequate duration of the test (until exhaustion) to obtain VO_2_ max [24], as well as an adaptation based on the subject’s training level. Therefore, using a start with different loads, all the groups would face tests of a similar duration and with the same increase in intensity [25]. The test was carried out on a cycle ergometer because of the greater accessibility for the collection of blood samples during the trial.

Training intensity and volume were reduced during the two previous days applying a regenerative load to avoid fatigue in the test.

### Sample collection

#### Blood samples

After a fasting period of 8 h and before the test, 5 mL of venous blood were extracted from the antecubital vein of each participant using a plastic syringe fitted with a stainless-steel needle. Once extracted, the samples were collected into a metal-free polypropylene tube (previously washed with diluted nitric acid) with EDTA as anticoagulant. The blood samples were immediately centrifuged for 10 min at 3000 rpm. The plasma was separated, and the erythrocytes were washed with 0.9% sodium chloride (NaCl) three times. The erythrocytes were aliquoted into Eppendorf tubes (previously washed with diluted nitric acid) and conserved at − 80 °C until biochemical analysis.

##### Determination of hematocrit and hemoglobin

The hematocrits were obtained by centrifuging the whole blood into a glass capillary containing heparin in a Microcen microfuge (Alresa. Spain). Hemoglobin (Hb) was determined using a Hb analyzer (HemoCue. Sweden).

#### Erythrocyte element determination

##### Sample preparation

The analysis was performed using inductively coupled plasma mass spectrometry (ICP-MS). To prepare the analysis, the decomposition of the organic matrix was achieved by heating it for 10 h at 90 °C after the addition of 0.8 mL HNO_3_ and 0.4 mL H_2_O_2_ to 2 mL of serum samples. The samples were then dried at 200 °C on a hot plate. Sample reconstitution was carried out by adding 0.5 mL of nitric acid, 10 μL of Indium (In) (10 mg/L) as an internal standard, and ultrapure water to complete 10 mL.

##### Standard and reference material preparation

Reagent blanks, element standards and certified reference material (Seronorm, lot 0511545, Sero AS Billingstand, Norway) were prepared identically and used for accuracy testing. Before the analysis, the commercial control materials were diluted according to the recommendation of the manufacturer.

##### Sample analysis

Digested solutions were assayed with an ICP-MS Nexion model 300D (PerkinElmer, Inc., Shelton, CT, USA) equipped with a triple quadrupole mass detector and a reaction cell/collision device that allows operation in three modes: without reaction gas (STD); by kinetic energy discrimination (KED) with helium as the collision gas; and in reaction mode (DRC) with ammonia as the reaction gas. Both collision and reaction gases such as plasmatic argon had a purity of 99.999% and were supplied by Praxair (Madrid, Spain). Two mass flow controllers regulated gas flows. The frequency of the generator was free-swinging and worked at 40 Mhz. Three replicates were analyzed per sample. The sample quantifications were performed with indium (In) as an internal standard. The values of the standard materials of each element (10 μg/L) used for quality controls were in agreement with intro and inter-assay variation coefficients of less than 5%.

### Statistical evaluations

Statistical analyses were carried out with the SPSS 20.0 for Windows. The results are expressed as *x* ± *s*, where *x* is the mean values and *s* the standard deviation.

The Dixon Q test was used to identify outliers. These values were analyzed to evaluate if their magnitude advised their elimination from the analyses. An exploration of the different variables was then carried out to determine normality, using the Shapiro-Wilks test, recommended for samples of less than 30 individuals. Subsequently, a comparison of the behavior of the variables among the three groups was made, using an ANOVA test, and a Bonferroni test was performed later on if there was significance.

A Pearson correlation study was carried out to ascertain if there was a relationship between erythrocyte changes in the concentrations of the elements and physical training. A significant difference was considered when *p* < 0.05.

## Results

Table 1 shows the anthropometric data of CG, MTG and HTG. As can be observed, the significantly decreased total weight and body fat percentage in MTG and HTG, indicate the adaptive consequences of training. The results of some ergospirometric parameters are also shown. A significant increase can be observed in both training groups, as would be expected. The data correspond to high endurance intensity trained athletes and subjects with a normal physical condition. Maximal VO_2_ and VE were significantly higher in the two training groups than controls. Maximal HR was lower in the controls than the training groups, and basal HR was lower in the training groups than controls.

**Table 1:** Characteristics of the three groups in the study

	CG (***n*** = 30)	MTG (***n*** = 24)	HTG (***n*** = 22)
**Height (m)**	1.75 ± 0.18	1.77 ± 0.05	1.73 ± 0.07
**Weight (kg)**	75.34 ± 12.03	75.12 ± 8.16	65.61 ± 5.22^†*^
**Σ6 skinfolds (mm)**	110.61 ± 34.29	87.78 ± 30.14	56.32 ± 16.65^†*^
**VO**_**2Máx**_**(mL**·**min**^**− 1**^·**kg**^**− 1**^**)**	38.90 ± 3.73	46.77 ± 6.72^++^	61.23 ± 3.00^††**^
**VE**_**max**_	89.33 ± 12.31	121.11 ± 21.40^++^	153.17 ± 15.84^††**^
**HR**_**rest**_**(beats**·**min**^**− 1**^**)**	72.58 ± 7.38	54.90 ± 10.85^++^	62.85 ± 7.57^††**^
**HRmax (beats**·**min**^**− 1**^**)**	184.32 ± 12.71	194.96 ± 8.3^+^	196.71 ± 6.21^†^

ANOVA and Bonferroni tests

† Differences between the HTG and CG (†*p* < 0,05; ††*p* < 0,01; †††*p* < 0,001)

+ Differences between the MTG and CG (+*p* < 0,05; ++*p* < 0,01; **+++***p* < 0,001)

* Differences between the HTG and MTG (*p < 0,05; **p < 0,01; ***p < 0,001)

Table 2 shows the daily intake of trace elements of all participants, showing that no significant differences were found among groups in the intake of any trace elements.

**Table 2:** Daily intake of Cr, Cu, Mn, Mo, Se and Zn in CG and sportsmen classified by the level of training

	CG (***n*** = 30)	MTG (***n*** = 24)	HTG (***n*** = 22)
**Cr (50–200** μg/d**)**	94.50 ± 52.72	97.23 ± 55.41	97.63 ± 60.55
**Cu (2000–3000** μg/d**)**	1843.5 ± 630.5	1799.2 ± 629.4	1785.3 ± 612.8
**Mn (2500–5000 μ**g/d**)**	3054.8 ± 1185.7	3115.4 ± 1275.3	3156.7 ± 1280.5
**Mo (75–400 μg/d)**	223.31 ± 184.33	225.0 ± 165.4	232.24 ± 156.1
**Se (50–200 μg/d)**	101.8 ± 17.25	102.4 ± 18.54	98.31 ± 17.85
**Zn (10–15 mg/d)**	14.23 ± 6.54	12.75 ± 6.48	12.85 ± 6.94

Table 3, shows the results of hemoglobin and hematocrit. Both parameters were similar in the three groups.

**Table 3:** Concentration of hemoglobin and hematocrit in the control group and sportsmen classified by the level of training

	CG (***n*** = 30)	MTG (***n*** = 24)	HTG (***n*** = 22)
**Hemoglobin (g/dL)**	14.97 ± 1.01	15.46 ± 1.45	15.14 ± 0.84
**Hematocrit (%)**	41.31 ± 1.18	42.89 ± 1.12	43.32 ± 1.17

ANOVA and Bonferroni tests

† Differences between the HTG and CG (†*p* < 0,05; ††*p* < 0,01; †††*p* < 0,001)

+ Differences between the MTG and CG (+*p* < 0,05; ++*p* < 0,01; **+++***p* < 0,001)

* Differences between the HTG and MTG (*p < 0,05; **p < 0,01; ***p < 0,001)

Table 4, shows erythrocyte concentrations of each metal. The results are expressed in μg/gHb, given that the major protein in the erythrocyte is hemoglobin and thus the results obtained in all cases were more solid.

**Table 4:** Erythrocyte concentration of the elements in the control group and sportsmen classified by the level of training

	CG (***n*** = 30)	MTG (***n*** = 24)	HTG (***n*** = 22)
**Cr (**μg/gHb**)**	0.32 ± 0.16	0.25 ± 0.14	0.12 ± 0.11
**Cu (**μg/gHb**)**	38.8 ± 6.68	31.81 ± 7.47^+^	30.24 ± 4.81^†††^
**Mn (**μg/gHb**)**	1.45 ± 0.36	1.22 ± 0.17^++^	1.16 ± 0.29^††^
**Mo (**μg/gHb**)**	0.3 ± 0.04	0.11 ± 0.16^+^	0.08 ± 0.02^†*^
**Se (**μg/gHb**)**	8.63 ± 2.30	7.99 ± 2.16	7.55 ± 1.36^†^
**Zn (**mg/gHb**)**	0.65 ± 0.10	0.49 ± 0.14^++^	0.44 ± 0.06^†††^

ANOVA and Bonferroni tests

† Differences between the HTG and CG (†*p* < 0,05; ††*p* < 0,01; †††*p* < 0,001)

+ Differences between the MTG and CG (+*p* < 0,05; ++*p* < 0,01; **+++***p* < 0,001)

* Differences between the HTG and MTG (*p < 0,05; **p < 0,01; ***p < 0,001)

When comparing concentrations between CG and MTG, Cu (*p* < 0.05), Mn (*p* < 0.01), Mo (*p* < 0.05) and Zn (*p* < 0.01) concentrations were lower in MTG. When comparing concentrations between CG and HTG, Cu (*p* < 0.001), Mn (*p* < 0.01), Mo (*p* < 0.05), Se (*p* < 0.05) and Zn (*p* < 0.001) were lower in HTG. MTG presented a higher concentration of Mo than HTG (*p* < 0.05).

Table 5 shows the correlations between the trace elements and training. Results are expressed with a correlation coefficient (**r**) and with a significance level (**p**). We found that the erythrocyte concentrations of Cu, Mn, Se and Zn were correlated with training.

**Table 5:** Correlations among the 76 subjects, represented by the r; statistical significance, between the essential trace elements and the level of training

Element	Training status
**Cr**	−0.164; *p* = 0.175
**Cu**	−0.790; *p* = 0.000
**Mn**	−0.422; *p* = 0.000
**Mo**	−0.081; *p* = 0.100
**Se**	−0.275; *p* = 0.021
**Zn**	−0.678; *p* = 0.000

## Discussion

This study found low erythrocyte concentrations in trace minerals of great importance for cellular functions in the subjects who trained. Cr is an essential trace element related to the metabolism of carbohydrates [26], altering the levels of blood glucose, potentiating the action of insulin, and influencing the metabolism of carbohydrates, lipids and protein anabolism [27].

It is possible that the mechanism by which exercise improves the response to insulin is related to an alteration in the metabolism of Cr. Thus, an acute exercise increases the urinary losses of Cr [28]. Clarkson (1991), indicates that little Cr is consumed in the general population, which suggests that athletes may have a deficit in this element [29]. However, Maynar et al., 2018 showed that serum concentrations in different sports modalities were significantly higher in athletes than in the control group [30].

In the present study, there were no significant differences in the concentrations among the three study groups in erythrocytes, and there was no correlation with the level of training, although the concentrations in the erythrocytes of athletes were significantly lower than in the control group.

The content of Cu in the red cells (0.79 ± 0.07 mg/L) was similar to those found by Lu et al., (2015) with a similar technique (erythrocyte non-Ficoll measurement) in Swedish subjects [31]. In the erythrocyte, almost 60% of Cu is linked to Cu-Zn SOD, which has been proposed as one of the best indexes to evaluate the body’s status regarding this element.

Studies that examine the effects of intense acute exercise on Cu levels show different results [32, 33]. Women who run a marathon increase their plasma levels after it without changes at the erythrocyte level [34]. In another study, running a marathon produces small increases in the plasma concentration of Cu, but at the same time, a decrease in the level in the whole blood [33]. Maynar et al. (2018) found similar serum concentrations in athletes of aerobic and anaerobic modalities to the control group, and only found levels significantly higher than the control group and the other two groups of athletes in soccer players (aerobic-anaerobic) [30].

In the present study, lower concentrations of Cu were found in the erythrocytes of professional athletes compared to the other groups, presenting a high negative correlation (*r* = − 0.790, *p* < 0.001) with the degree of training. It is known that the erythrocyte contains the enzyme Cu-Zn-SOD and that according to the study by Mena et al. (1991) its enzymatic activity is higher in amateur and professional cyclists with respect to sedentary subjects, but it is also observed that Cu-Zn-SOD decreases its activity the greater the level of training of the cyclist [35].

In theory, the function of copper enzymes is essential for physical performance. For example, mitochondrial cytochrome-c oxidase catalyzes the final step in the aerobic respiratory chain. Also, three copper enzymes (ceruloplasmin, cytosolic superoxide dismutase, and extracellular superoxide dismutase) have essential antioxidant functions that can reduce the free radicals formed during physical activity that would produce oxidative stress and could lead to fatigue and delay in muscle recovery [36].

Mn is a trace element present in all tissues of terrestrial and aquatic organisms. Its average concentration in erythrocytes is 22 ± 7.4 μg/L as measured by ICP-MS (erythrocyte non-Ficoll measurement) [31]. It is a component of several enzymes, including arginase, glutamine synthetase, phosphoenolpyruvate decarboxylase, hexokinase, xanthine oxidase and Mn-SOD. The glycosyltransferases and xylosyltransferases, which are involved in the synthesis of proteoglycans and therefore in the formation of bone, are sensitive to Mn.

Several studies have observed that physical exercise increases the activity of Mn-SOD, which could be related to changes in serum concentrations of this element [37]. This is significant because Mn-SOD is an antioxidant enzyme located at the level of the mitochondria that would neutralize the superoxide radicals that form inside it. Therefore, it is suggested that the practice of physical exercise and the consequent increase in the activity of Mn-SOD can induce cardioprotection and in general protection of cellular mitochondria against free radicals (Reactive Oxygen Species related to damaging cells, lipids and DNA) produced by sports practice [38].

But in addition to the Mn-SOD, Mn is part of enzymes that are fundamental in gluconeogenesis, and this is important especially in athletes of aerobic physical activities and also in the formation of urea, which is crucially important in aerobic physical activities [39]. Maynar et al. (2018) found that Mn in serum was much higher in aerobic than in sedentary people. However, in athletes with a greater anaerobic component, these concentrations were lower, even compared to the control group [30]. However, we have not found studies that tell us about the values at the cellular level in athletes.

In our study, we found for Mn something similar to that seen for Cu, that is, lower concentrations of Mn in the erythrocytes of professional athletes compared to the other groups, presenting a high negative correlation (*r* = − 422; *p* < 0.001) with the degree of training. However, unlike Cu, there is no Mn-SOD inside the erythrocyte because there are no mitochondria in the erythrocyte, so the lower concentration of this element would probably be due to a smaller amount of this element in the body of the athlete, which manifests itself in the erythrocyte, that could act as a reservoir.

The concentrations of Mo in erythrocytes are 0.52 ± 0.28 measured by ICP-MS [31]. Mo functions as an enzymatic cofactor of three enzymes (aldehyde oxidase, sulfite oxidase, and xanthine dehydrogenase), which catalyze the hydroxylation of several substrates [40]. The aldehyde oxidase oxidizes and detoxifies several pyrimidines, purines and pteridines and related compounds. Thus, sulfite oxidase catalyzes the transformation of sulfite to sulfate, from cysteine and methionine or directly from the diet. Xanthine dehydrogenase catalyzes the conversion of hypoxanthine to xanthine and from xanthine to uric acid. Therefore, molybdenum is involved in the purine cycle and the final production of uric acid, considered an antioxidant in the human body [41].

The higher concentrations of molybdenum would facilitate the formation of uric acid and thus avoid the damage that superoxide anions would produce generated by xanthine oxidase in the ischemia-reperfusion processes that occur in athletes’ muscles during intense activity [40].

In this study, we found significantly higher concentrations of Mo in the MTG and HTG group than in the controls. However, there was no correlation between this element and the training level of the subjects, which indicates that the erythrocyte changes of the element would not be related to the training.

Blood Se, which represents about 3% of total body Se [42, 43], is distributed in plasma, red blood cells and white blood cells. The erythrocyte values of Se, with ICP-MS, are 0.15 ± 0.03 mg / L [31]. In red blood cells Se, as in other cells, prevents the deterioration of the membrane cytoskeleton [8]. Its initial role in these cells is to protect the hemoglobin from oxidation. Also, it is well established that most of the Se is bound to hemoglobin, and a small part is in glutathione peroxidase (GSH-Px), the main action of which is to protect the body from the cytotoxic activity of hydroperoxides and free radicals, since they prevent the formation of lipid peroxides in the cell membrane [44]. Therefore, it has antioxidant properties and plays an essential role in the defense against free radicals, which occurs largely in situations of trauma and overexertion, as well as during strenuous exercise.

Contrary to Cu and Zn, the variations in erythrocyte Se are strongly dependent on dietary habits [45]. In a state of equilibrium, there is a close relationship between the erythrocyte and plasmatic Se [46].

Pograjc et al. (2012) found that at the end of the training period of the soldiers in their study, the level of Se in whole blood decreased, but that of plasma did not change or increased slightly [47]. This could be the result of a decrease in Se concentration in erythrocytes, but not at the expense of a reduction in the activity of GSH-Px. So, there must be another form of storing interchangeable Se in erythrocytes. The decrease in total blood concentrations could indicate that some of the erythrocyte Se would be transferred to the tissues during training [47].

In the study by Mena et al., (1991), an increase in GSH-Px activity was observed in the erythrocytes of professional cyclists, keeping this relationship with the degree of training [35]. Therefore, something similar to that indicated by Pograjc et al. (2012) must have happened in our study where there was a significant correlation of Se with the degree of training (*r* = − 275, *p* < 0.05) accompanied by decreases in the athletes’ erythrocytes, although without reaching statistical significance. This, together with the fact that the values found in all the groups were lower than those found by Lu et al. (2015) in their study in non-athlete subjects, would indicate a possible deficiency of the element in our subjects that, given its low levels and its importance in the organism, would prevent further decreases.

In erythrocytes, Zn is a cofactor of carbonic anhydrases, SOD, and 8-aminolevulinate dehydratase. In addition, zinc is bound to the erythrocytic membrane, hemoglobin, and other proteins, as well as to small molecules [48]. So far, contradictory reports have been presented regarding erythrocyte Zn in physically active subjects [34, 49, 50] so that no firm conclusions can be drawn. In trained athletes erythrocyte values of Zn are lower [34]. Some articles hypothesize about redistribution in tissues [51] and most refer to poor homeostatic control of Zn in red blood cells. Some studies report that Zn increases in plasma and decreases in erythrocytes after intense running on a treadmill or an extraordinary effort on a cycle ergometer. These studies propose that such plasma increases are due to a Zn leakage from muscles damaged by physical activity [52].

It can be assumed that functional displacements of Zn can occur between tissues during exercise, as occurred with Se. For that reason, it seems quite difficult to determine the effects of exercise on the concentration of Zn and other elements [53].

In the present study, the erythrocyte concentrations of Zn showed a high negative correlation with the degree of training (*r* = − 0.678, *p* = 0.000), which would indicate that they are dependent on sport training. Their level in those who practiced physical exercise was lower than those in the control group, being the lowest in the athletes with the highest degree of training.

This could indicate that our athletes could present a deficit in their nutritional status as indicated by De Carvalho et al. (2012) in their study, performed on swimmers. These authors observed plasma Zn concentrations below normal levels and erythrocytic Zn levels at borderline or within the lower reference limits. The low or limiting levels of this mineral, since before initiating training, suggest that the athletes started the training phases with existing deficiencies pointing to the need to re-evaluate the Zn requirements of athletes [54].

This possible deficiency, similar to that of the other essential elements, could have an important impact on an athlete’s performance, which might be significantly reduced in different aspects as seen above.

## Conclusions

In conclusion, our study shows a deficiency in Cu, Mn and Zn in subjects who perform physical training, which does not exist in subjects who do not engage in training, and these deficiencies are correlated with sport training. Therefore, we believe that the erythrocyte evaluation of these elements should be carried out on athletes who regularly perform physical activity before and during their training phase. We also think that more studies are needed in all the elements studied in erythrocytes and if possible, in different types of athletes, because if these data were confirmed, products containing these elements would have to be included in the diets of these athletes during periods of training and competition.

Previous studies have reported low serum concentrations of several trace metals in athletes from different modalities, like Mn in anaerobic athletes or Zn in aerobic and anaerobic athletes [18, 30]. These results could indicate the need for extra supplementation. However, low seric values are not sure signals of deficits, since if the concentrations are high in the cells (a possible storage place), a deficit may not be certain. So, the most important element of the current study was to determine the erythrocyte concentrations of these elements and to compare them with serum or plasma values, to ascertain possible deficits, in which case, supplementation could be suggested.

## Data Availability

All data generated or analyzed during this study are included in this published article.

## Abbreviations

Cr: Chromium
Cu: Copper
Mn: Manganese
Mo: Molybdenum
Se: Selenium
Zn: Zinc
MTG: Moderate training group
HTG: High training group
CG: Control group
ICP-MS: Inductively coupled plasma mass spectrometry
Hb: Hemoglobin
Σ6: Sum of 6 skinfolds
VO_2_: Oxygen Consumption
VE: Expiratory Volume
HR: Heart Rate
SOD: Superoxide dismutase
H_2_O_2_: Hydrogen peroxide
GSH-Px: Glutathione peroxidase
