# Correction to: Erythrocyte concentrations of chromium, copper, manganese, molybdenum, selenium and zinc in subjects with different physical training levels

**DOI:** 10.1186/s12970-021-00428-2

**Published:** 2021-04-14

**Authors:** M. Maynar, F. J. Grijota, J. Siquier-Coll, I. Bartolome, M. C. Robles, D. Muñoz

**Affiliations:** 1 School of Sport Sciences, University of Extremadura, University Avenue, Avenida de la Universidad s/n, 10003 Cáceres, Spain; 2 Faculty of Language and Education, Antonio de Nebrija University, Madrid, Spain

**Correction to: J Int Soc Sports Nutr 17, 35 (2020)**

**https://doi.org/10.1186/s12970-020-00367-4**

The original article [1] contains an incorrect affiliation for co-author, F. J. Grijota.

The correct affiliation can be viewed in this correction article (Faculty of Language and Education, Antonio de Nebrija University, Madrid, Spain).
